# Mating Type Gene Homologues and Putative Sex Pheromone-Sensing Pathway in Arbuscular Mycorrhizal Fungi, a Presumably Asexual Plant Root Symbiont 

**DOI:** 10.1371/journal.pone.0080729

**Published:** 2013-11-19

**Authors:** Sébastien Halary, Laurence Daubois, Yves Terrat, Sabrina Ellenberger, Johannes Wöstemeyer, Mohamed Hijri

**Affiliations:** 1 Département de Sciences Biologiques, Institut de Recherche en Biologie Végétale (IRBV), Université de Montréal, Montréal, Québec, Canada; 2 Institute of General Microbiology and Microbe Genetics, Friedrich-Schiller-Universität Jena, Jena, Germany; Nanjing Agricultural University, China

## Abstract

The fungal kingdom displays a fascinating diversity of sex-determination systems. Recent advances in genomics provide insights into the molecular mechanisms of sex, mating type determination, and evolution of sexual reproduction in many fungal species in both ancient and modern phylogenetic lineages. All major fungal groups have evolved sexual differentiation and recombination pathways. However, sexuality is unknown in arbuscular mycorrhizal fungi (AMF) of the phylum Glomeromycota, an ecologically vital group of obligate plant root symbionts. AMF are commonly considered an ancient asexual lineage dating back to the Ordovician, approximately 460 M years ago. In this study, we used genomic and transcriptomic surveys of several AMF species to demonstrate the presence of conserved putative sex pheromone-sensing mitogen-activated protein (MAP) kinases, comparable to those described in Ascomycota and Basidiomycota. We also find genes for high mobility group (HMG) transcription factors, homologous to *SexM* and *SexP* genes in the Mucorales. The *SexM* genes show a remarkable sequence diversity among multiple copies in the genome, while only a single *SexP* sequence was detected in some isolates of *Rhizophagus irregularis*. In the Mucorales and Microsporidia, the *sexM* gene is flanked by genes for a triosephosphate transporter (TPT) and a RNA helicase, but we find no evidence for synteny in the vicinity of the *Sex* locus in AMF. Nonetheless, our results, together with previous observations on meiotic machinery, suggest that AMF could undergo a complete sexual reproduction cycle.

## Introduction

Sexual reproduction drives natural selection and adaptation of organisms by generating genetic diversity through recombination. This allows beneficial mutations to spread, and purges deleterious mutations in eukaryotic genomes [1,2]. However, many eukaryotic organisms rely occasionally or sometimes exclusively on clonal propagation, fueling the on-going debate about advantages versus costs of sexual reproduction [3,4]. In fungi, mitotic propagation via sporangiospores and conidia is often a much more efficient means of propagation than meiotic processes requiring spore formation. A perfect illustration of this is given by the members of arbuscular mycorrhizal fungi (AMF; phylum Glomeromycota) lineage, which are believed to reproduce exclusively clonally via the production of multinucleate spores that contain hundreds of spores [5,6].

Arbuscular mycorrhizal fungi are ecologically crucial symbionts that enhance uptake of plant nutrients, especially phosphate, as well as protecting plants against pathogens [7,8]. AMF have been functionally associated with land plants since the Devonian, approximately 450 M years ago, and it has been suggested that they belong to the intriguing club of ancient asexual organisms [9]. However, a recent study has challenged this view by showing that AMF have conserved a set of genes encoding the complete meiotic machinery [10]. These findings suggest that AMF could undergo meiotic recombination events, even though the existence of a cryptic sexual cycle still remains to be demonstrated. Indeed, only one author has described multinuclear propagation structures, in *Gigaspora decipiens*, which are different from generally observed chlamydospores, that could cautiously be interpreted as products of putative sexual development [11,12].

In the fungal kingdom, sexual reproduction is controlled by mating type loci [13,14]. Two basic categories of sexual differentiation are distinguished: homothallism (self-fertile or inbreeding) and heterothallism (outbreeding). In homothallism, the information for both mating types is found either in the same nucleus (primary homothallism) or in two different nuclei within the same cell (secondary or pseudo-homothallism). Sexual reproduction in both yeasts and filamentous fungi starts with partner recognition mediated by mating type-specific pheromones and receptors [13,14]. After mate-recognition, cell fusion occurs, followed by nuclear fusion and meiotic recombination [13,14]. Despite the great diversity of mating modes in fungi, pheromone-receptor systems and subsequent signal transduction pathways share considerable similarities, and transcription factors related to mating type loci appear to be ubiquitous and highly conserved. 

In Asco- and Basidiomycota, two phyla of the fungal subkingdom Dikarya, essential parts of the pheromone-sensing pathway are highly conserved [15,16] The core set of sex pheromone-sensing pathway genes encodes proteins involved in signal transduction between pheromone receptors at the hyphal surface and the transcription factors responsible for regulating the mating process. The sex pheromone-sensing pathway in *Saccharomyces cerevisiae* is well documented in many literature reviews [14,17]. Briefly, in *S. cerevisiae*, this pathway is activated by binding of a short peptide pheromone to the membrane-bound pheromone receptor (STE2 or STE3). This interaction induces dissociation of a receptor-coupled heterotrimeric G-protein (GPA1, STE4, STE18). Subsequently, the STE4-STE18 dimer activates the STE20 Pak kinase and recruits the scaffolding protein STE5 with its associated MAP kinase proteins (STE11, STE7 and FUS3). The cascade is initiated when STE11 is phosphorylated by STE20, which is coupled to the adaptor protein STE50, and ends by the activation of FUS3, that triggers the mating response by phosphorylating several targets responsible for arresting the cell cycle, and for allowing polarized growth and cell fusion, such as STE12. Up to date, little is known about the sex-related signal transduction in other fungal phyla, but it is thought to be driven by completely different pathways. For instance, in the phylogenetically basal fungal order of Mucorales, sexual communication is mediated by trisporic acids, β-carotene derived molecules of the trisporoids family [18]. 

In contrast to animals and some plants, where transcription factor genes typically located on sex-specific chromosomes control sexual determination, in fungi, sex or mating type determination is controlled by a small specialized region of the genome, designated the *Mat* or *Sex* locus [14,19]. Sex loci were first characterized in Dikarya [19]. Later Idnurm et al., (2008) [20] described, at the molecular level, a comparable *Sex* locus in the mucoralean zygomycete Phycomyces *blakesleeanus* which had previously been identified by classical genetics. These loci have been found in other Mucorales [21], and interestingly, in Microsporidia [20,22,23], a group of obligate intracellular parasites that seems to be phylogenetically related to zygomycetes. In Microsporidia as well as in Mucorales, the *Sex* locus is a cluster of three genes with conserved synteny. In Mucorales these loci control the sexual differentiation processes via high mobility group (HMG) transcription-factor genes, flanked by triose-phosphate transporter (TPT) and RNA helicase genes [20-22]. In Microsporidia, for which sexual processes have not been observed, these loci are possibly an indication that there is an extant sexual life style [22]. To assess the hypothesis of a sexual cycle in AMF, we performed a genomic and transcriptomic survey of the AMF model species *Rhizophagus irregularis* using the publicly available expressed sequence tags (ESTs) [24] and a genomic survey in *Rhizophagus* *sp.* and *Glomus cerebriforme*. We found evidence for a putative pheromone-sensing pathway as well as for transcription factor genes in AMF that are very similar to those determining mating types in related fungi.

## Material and Methods

### Fungal strain and growth conditions

Isolates of *Rhizophagus irregularis* (Synonym, *Glomus irregulare*; formerly *Glomus intraradices*)*, Rhizophagus* *sp.* and *Glomus cerebriforme* were obtained from the fungal collection of the Department of Agriculture DAOM (Ottawa, ON, Canada). Isolate reference numbers were 197198, 234179, 234328, 240415, 240429, 240159 and 213198 for *R. irregularis*; 227022 for *G. cerebriforme* and 229456 for *Rhizophagus* *sp*. Fungal isolates were cultured *in vitro* with Ri-T-DNA transformed *Daucus carota* roots in Petri dishes containing minimal medium (M) solidified with 0.4 % Gellan gum (Sigma Aldrich, Canada). The cultures were kept in an incubator at 25 °C in the dark. An AMF isolate is a culture that was originally started from a single spore and subcultured by transferring 1 to 2 cm^2^ of gel containing a mixture of mycorrhized roots, spores and hyphae to a new Petri dish.

### Extraction and pyrosequencing of DNA from AMF

Spores and hyphae were harvested by dissolving the Gellan gum matrix in which cultures were grown in a solution containing 0.0083 N sodium citrate and 0.0017 N citric acid. Spores and hyphae were gently crushed in a 1.5 ml microtube using a sterilized pestle. DNA was extracted using the DNeasy Plant Mini kit (Qiagen), according to the manufacturer’s instructions. The purified DNA was sent to the Genome Quebec Innovation Centre (McGill University, Montreal) for pyrosequencing using the GS FLX Titanium whole genome shotgun kit (Roche 454 Life Sciences), employing a full run for each DNA sample with an average coverage of 3X. Reads were quality trimmed using LUCY (Chou, 2001) through the lucyTrim.pl script from OCTOPUS (http://octupus.sourceforge.net). *De novo* DNA sequence assemblies were performed (CLCbio, http://www.clcbio.com/).

### *In silico* identification of genes for the pheromone sensing pathway and *Sex* loci

Orthologues of 12 genes coding for proteins involved in the pheromone-sensing pathway of *Saccharomyces cerevisiae* were searched across the genomes of *Rhizophagus* spp., *G. cerebriforme* and the publicly available expressed sequence tags (ESTs) from *G. irregulare* [24], using TBLASTN. The TPT and the RNA helicase genes of the *Sex* cluster of *Rhizopus* spp., *Phycomyces blakesleeanus* and *Mucor mucedo* were also used as query sequences. Finally, mating type genes of a broad range of fungal taxa, including Ascomycota, Basidiomyceta and Mucorales, were searched in the same way. Orthology of the AMF genes was first assessed using the best-reciprocity approach. Each resulting best hit, exhibiting a cut off e-value lower than 1e^-05^, was aligned with all proteins of *Saccharomyces cerevisiae* or *P. blakesleeanus* and *M. mucedo* using BLASTX. Putative open reading frames of *Rhizophagus* spp. and *G. cerebriforme* candidate sequences were annotated by pairwise comparisons using BLASTX against GenBank sequences and multiple sequence alignments with orthologous gene families from InParanoid using MUSCLE v. 3.7 [25]. Pfam family and domain information were also retrieved from the Sanger Pfam server (http://pfam.sanger.ac.uk/). Finally, to confirm orthology (vs paralogy), *Rhizophagus* spp. and *G. cerebriforme* homologs were submitted to a phylogenetic comparison with homologous sequences of representative fungi and, when possible, with representatives of the Animalia as outgroups. Multiple amino acid sequence alignments (MUSCLE v. 3.7 [25]) were inspected and divergent or ambiguous positions were removed using BioEdit v.7 [26]. For each protein, evolutionary models were determined using ProtTest [27]. The phylogenetic trees were inferred by PhyML v3.0 [28], using the best model and 4γ with 1,000 bootstrap replicates.

All the nucleotide sequences were deposited in Genbank under the accession numbers: HF679491 - HF679525, HG421031, HG421032 and are shown in Table S1.

### *In silico* identification of TSP1 protein

Homologues of mucoralean genes involved in the trisporic acids biosynthesis (TSPs) were searched via protein domain comparisons. Global pairwise sequence alignments between the candidate protein from *Rhizophagus* and zygomycetous TSP1 proteins were created with the Needleman–Wunsch algorithm, implemented in EMBOSS (European Molecular Biology Open Software Suite [29]). Clustal Omega [30] implemented in the UniProt database, was used for multiple sequence alignments. We aligned the *Rhizophagus* spp. TSP1 candidate protein with known TSP1 proteins from *Mucor mucedo*, *Parasitella parasitica*, and *Rhizopus delemar*. Predictions about the tertiary structure of XYL2 were derived with 3DLigandSite [31], which determines a three-dimensional model structure of the query protein before it starts predictions for ligand-binding sites. 3DLigandSite uses Phyre2 [32] to predict the protein structure of the query sequence and to find suitable structural models already entered in the PDB.

We used Chimera [33] for structure alignments and visualization.

Fungal orthologs of *Rhizopus oryzae* TSP1 coding gene (RO3T_11715) were retrieved using InParanoid v. 3.0 [34]. Predictions of protein families and domains were identified using InterProScan [35]. To evaluate similarity between TSP1, *Rhizophagus* candidate and the other fungal proteins, a multiple alignment of all proteic sequences were obtained using COBALT [36], and a similarity matrix was obtained using BioEdit v.7 [26].

Here, D-xylose reductase was the best template for XYL2 (Protein Data Bank (PDB) ID: 1MI3_A; sequence identity with XYL2: 49%). This D-xylose reductase from *Candida tenuis* was also the best template for TSP1 from *Mucor mucedo* [37]. The structure of 1MI3_A achieves coverage of 97 % of the TSP1 sequence, sequence identity of 46 % and a confidence score of 100. 

The structure alignment of XYL2 and TSP1 has a RMSD of 0.177 Å. The structure alignments of TSP1 and XYL2 with the template XYL1 (PDB ID: 1K8C) result in the same RMSD of 0.348 Å. Also the Q-score of the structure alignment of XYL2 and TSP1 supports the assumption that XYL2 is a TSP1 protein. A Q-score of 1 stands for two identical structures; we obtained a value of 0.962 for this alignment.

**Figure 1:**
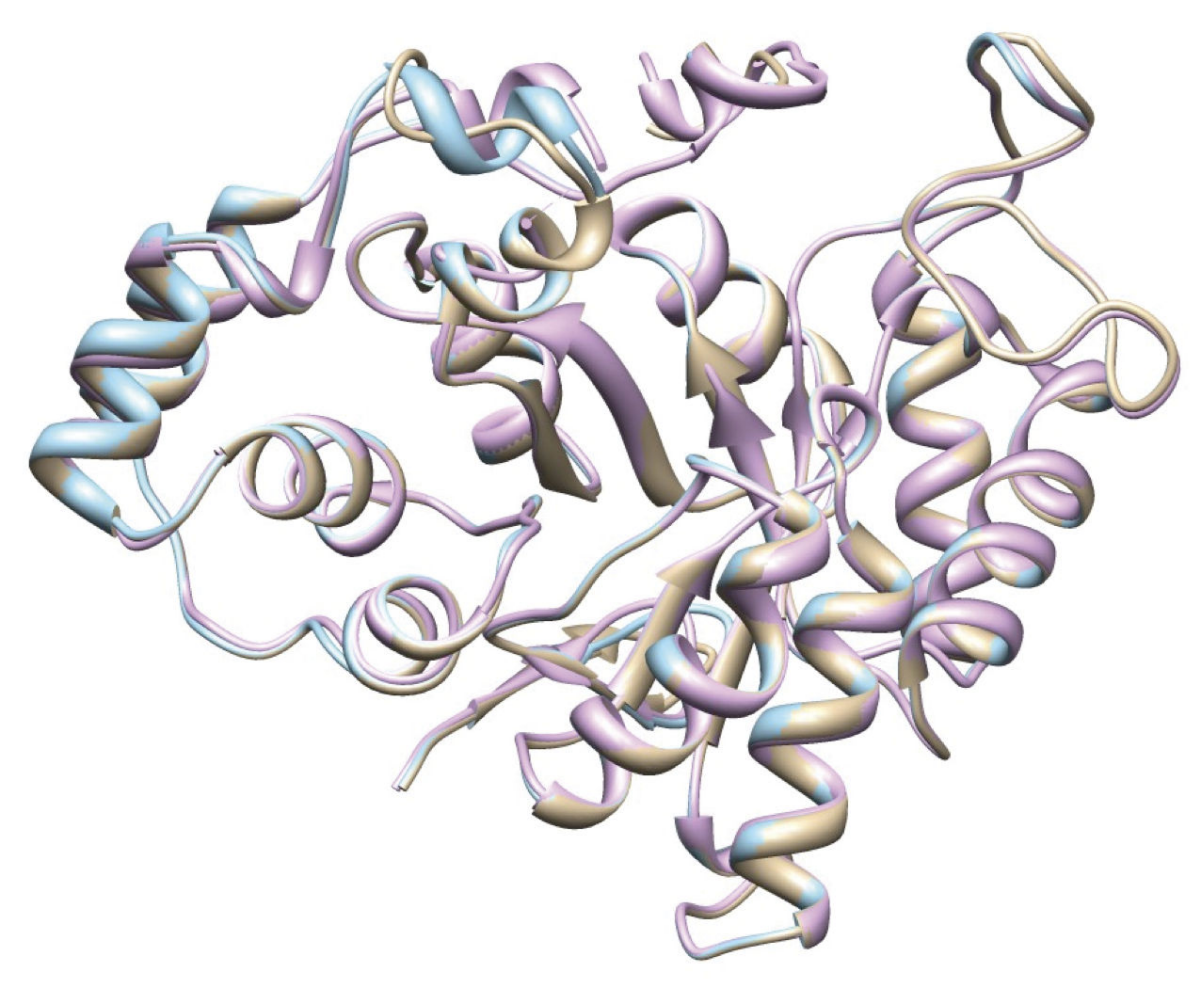
3D structure comparison of XYL1, XYL2 and TSP1. Structure alignment of XYL1 (pink), XYL2 (brown) and TSP1 (blue), showing that XYL protein shares all protein domains with TSP1, known to belong to the aldo/keto group of dehydrogenases.

Most of the amino acids for NADP binding are identical in XYL2 and TSP1 (Figure S1). They also have the same amino acids in their active sites (Figure S1), an aspartate, a tyrosine, a lysine, and a histidine. Structure alignment of XYL1, XYL2 and TSP1 shows high similarity between the three proteins (Figure 1, Video S1). 

### PCR amplification and Sanger sequencing

PCR was used to validate gene sequences retrieved from 454-genomic sequencing and EST data (Table S2). Degenerated primers were also used to identify genes that were not found in genomic and EST sequences. PCR reactions were performed using Taq DNA polymerase (Quiagen) following the manufacturer’s recommendations. PCRs were performed in a volume of 50 µl under the following conditions: denaturation at 95 °C for 2 min, followed by 35 cycles of 94 °C for 30 sec, temperature gradient between 48 °C to 54 °C (depending on the primers) for 30 sec and 70°C for 2 min. Final elongation was performed at 72 °C for 10 min. PCRs were run on a Mastercycler Pro S gradient thermocycler (Eppendorf, Canada). PCR amplicons were visualized on a 1% agarose gels stained with GelRed (Invitrogen, Canada). Successful PCR amplicons were extracted from gels, purified and sequenced according to the conventional Sanger technique at the Genome Quebec Innovation Center (Montreal, QC).

Long PCR was used to test synteny of *Sex* loci in *Rhizophagus* spp. Six primers were designed for the genes encoding TPT, SexM and RNA helicase (Table S2). All possible combinations of these primers were performed using the TaKaRa LA PCR Kit 2.1 (CloneTech) following the manufacturer’s recommendations. Long PCRs were performed in a volume of 50 µl under the following conditions: pre-denaturation at 95 °C for 5 min followed by 35 cycles of 95 °C for 30 sec, 48 °C for 30 sec and 72 °C up to 20 min. Final elongation was performed at 72 °C for 10 min. PCRs were run on a Mastercycler Pro S Gradient thermocycler (Eppendorf, Canada). PCR amplicons were visualized on 1 % agarose gels stained with GelRed (Invitrogen, Canada) 19 598 bp amplicon. PCR products for TPT, *SexM* and RNA helicase were validated by conventional Sanger DNA sequencing. 

### Estimation of SexM and SexP relative copy number

Quantification of *SexM* and *SexP* copy numbers was performed by a qPCR approach. The assay was developed for a conserved region using Primer Express Software Version 2 (Applied Biosystems ; Table S2). Probes were labelled with FAM at the 5’ end and BHQ-1 at the 3’ end. Fluorescence data were collected with the ViiA^TM^ 7 Real-Time PCR System (Applied Biosystems). qPCR was performed in 3 replicates with 6 dilutions per replicate. Copy numbers were established for each sample of target DNA using the single copy reference gene *Rad15* [38]. PCR efficiency was calculated by converting the slope, produced by the linear regression of the curves, to percentage efficiency using the formula: Efficiency = -1 + 10 ^(-1/slope)^.

## Results

### AMF pheromone-sensing pathway

**Figure 2:**
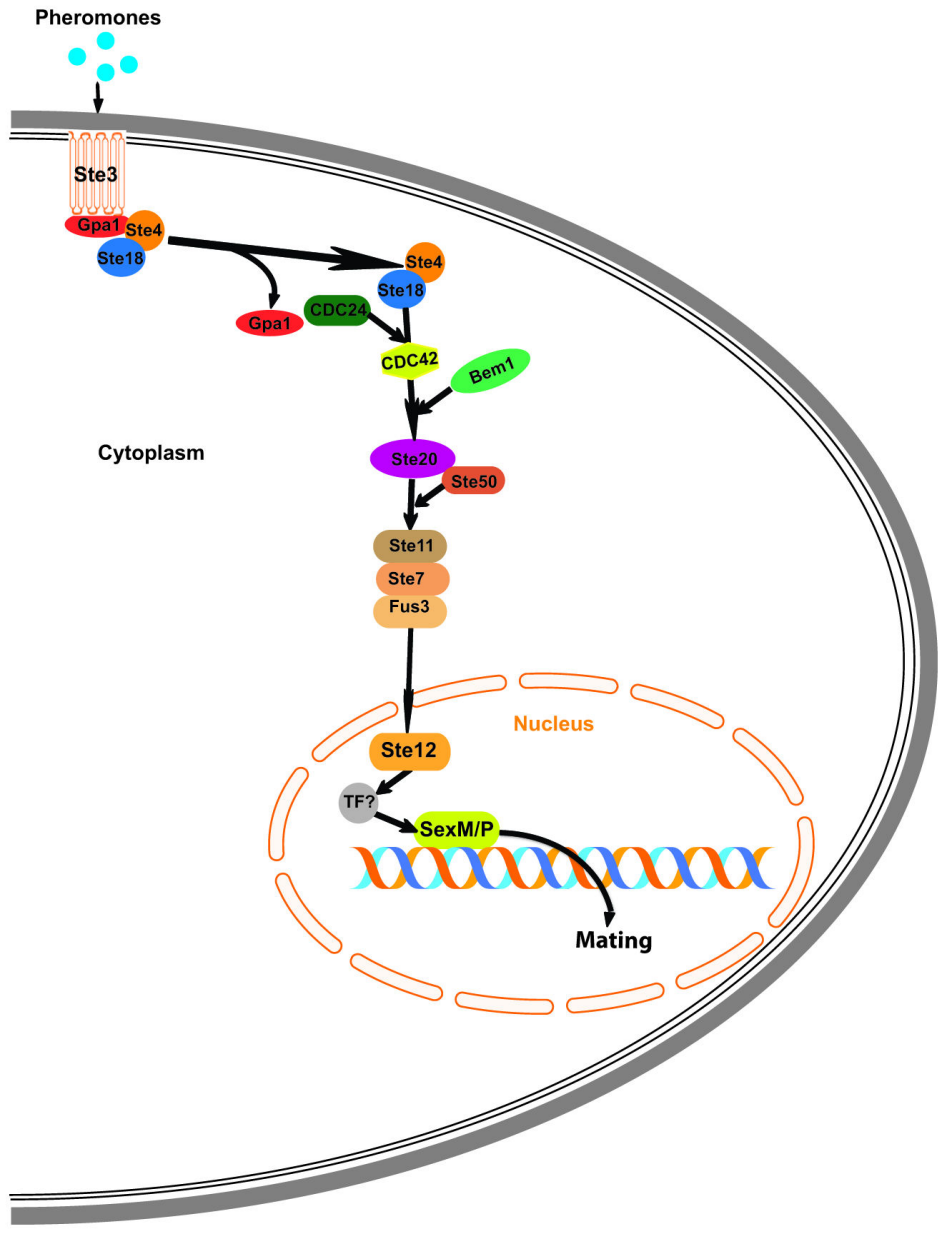
Hypothetical pheromone sensing pathway in *Rhizophagus irregularis*. A putative pheromone binds to its receptor (STE3) that is coupled to a heterotrimeric G-protein designated GPA1 (α-subunit), STE4 (β-subunit), and STE18 (γ-subunit). The putative pheromone binding to its receptor leads to G-protein activation and the dissociation of STE4 and STE18 from Gpa1. The released complex (STE4/STE18) activates STE20 that stimulates the MAPK module MKKK STE11, MKK STE7 and MAPK FUS3. The activated form of FUS3 may be translocated to the nucleus, where it activates STE12. STE12 induces putative transcriptional factors of the Pheromone Response Element that activates the *SexM* or *SexP* gene. Activation of STE 11 could also be achieved by an independent putative mechanism involving STE50 through the activation of STE20.

The genomes of *Rhizophagus* spp. and *G. cerebriforme* analyzed in this study possess orthologous genes coding for all the proteins in the *S. cerevisiae* sex pheromone-sensing pathway (Figure 2 and Table S3), except for the scaffolding STE5 gene. Furthermore, an ortholog of the ABC transporter STE6, responsible for secretion of the farnesylated pheromone a-factor in baker’s yeast, was also characterized. Only one gene copy for the STE3 ortholog or a-factor receptor was found by two approaches, genome survey and PCR with degenerate primers. However, no peptide pheromone coding genes related to a-factor or α-factor were found. Figure 2 shows a hypothetical pheromone-sensing pathway in *R. irregularis* that could be involved in signal transduction from pheromone-receptors at the hyphal surface, through MAP kinases, to *SexM/P* transcription factors responsible for regulating the mating process. All the genes involved in this pathway are putatively functional and are found in expressed sequence tags (ESTs) (Tisserant et al., 2012), except STE50. All the DNA sequences of these genes were validated using PCR, cloning and Sanger sequencing.

### Genes potentially involved in biosynthesis of fungal pheromones

No orthologous genes coding for peptide pheromones of Dikarya were found in our data set. Similarly, genes putatively encoding the TSPs cannot directly be detected by simple sequence similarity approaches. However, since TSPs contain specific protein domains, we also looked for proteins in *R. irregularis* sharing high similarity with TSPs domains from the model mucoralean fungus *Mucor mucedo*. By this approach, we identified in *R. irregularis* a putative TSP1-candidate in the UniProt database (www.uniprot.org) that was annotated as xylose reductase 2 (XYL2, UniProt ID: G8I0D9). At the level of 3D-structures, the TSP1 protein from *Mucor mucedo* [Ellenberger, 2013 #169] matches nearly completely the corresponding protein in *R. irregularis* (Figures 1 and S1; Video S1). However, InParanoid orthology prediction also allowed retrieval of mucoralean TSP1 orthologs in 12 ascomycotan and 1 basidiomycotan genomes. These fungi are not known to use trisporic acid-mediated communication. All these proteins, as well as *Rhizophagus* candidate, share protein family and domain signatures with TSP1, known to belong to the aldo/keto reductase group of dehydrogenases [39] (IPR001395. Aldo/ket_red., IPR018170. Aldo/ket_reductase_CS., IPR020471. Aldo/keto_reductase_subgr., IPR023210. NADP_OxRdtase_dom.). Furthermore, the *R. irregularis* candidate protein shares more similarity with 7 ascomycotan and the basidiomycotan sequences than with *Rhizopus* TSP1 protein (Table S4).

### Genes for mating control

**Figure 3:**
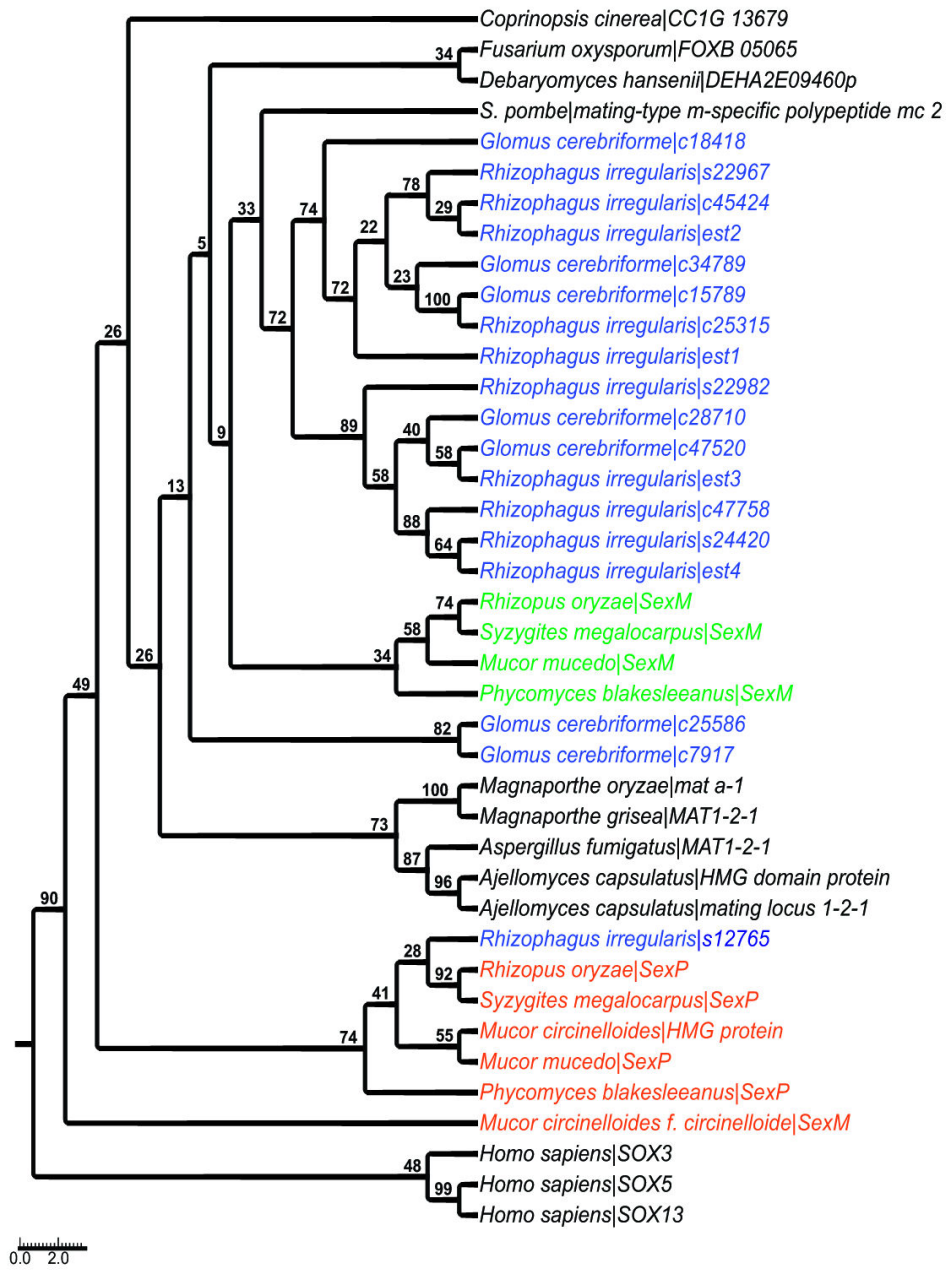
Phylogenetic tree of mating type transcription factors. Maximum likelihood of amino acid sequences of *SexM* and *SexP* analysed with the LG+G+F (with four distinct gamma categories) phylogenetic model of mating type HMG-box proteins. The *Rhizophagus* *spp.* sequences are highlighted in blue, mucoralean *SexM* sequences in green, and *SexP* in orange. The ascomycotan, basidiomycotan, as well as the human outgroup sequences are in black. The numbers at branches correspond to bootstrap support values generated with 1,000 bootstrap replicates. As previously described [21,56], internal nodes are not statistically supported due to high evolutionary rates of mating type proteins [57], leading to poorly supported topologies. However, the *R. irregularis* sequences s12765 and mucoralean *SexP* proteins fall into the same clade, while other *Rhizophagus* *spp.* sequences are grouped together in the star-shaped cluster with mucoralean *SexM* and other fungal domains mating type proteins.

We retrieved 17 sequence variants (alleles) of the minus type specific transcription factor gene *SexM*, 3 alleles and 4 mRNAs in the *R. irregularis* isolate DAOM-197198, 3 alleles in *R. irregularis* isolate DAOM-234179, and 7 alleles in *G. cerebriforme* genomes (Figure 3). While many different copies with considerable sequence diversity were found in the *SexM* gene, we retrieved only a single sequence of the plus type specific transcription factor gene *SexP* in the genome of *R. irregularis* isolate DAOM-197198. This unexpectedly biased situation led to the question of whether *SexP* occurs in genomes other than *R. irregularis* isolate DAOM-197198 and whether *SexM* and *SexP* genes occur in comparable copy numbers in *G. irregulare*. We thus screened 7 DAOM isolates of *R. irregularis* (197198, 234179, 234328, 240415, 240429, 240159 and 213198) by PCR, cloning and sequencing as well as by quantitative PCR. Because it turned out to be difficult to define an overall consensus sequence of the *SexM* gene, we removed some of the more divergent alleles in order to design primers for reasonably conserved parts of the gene. We amplified partial sequences of *SexM* (638 bp) and *SexP* (230 bp) from genomic DNA of the different *R. irregularis* DAOM isolates. *SexM* sequences revealed intraspecific polymorphisms ranging from 0.78 % and 2.67 % at the level of DNA sequence in all isolates. This is likely to represent an underestimation of intraspecific polymorphism of *SexM* sequences, because we targeted only some sequences, i.e. those that we were able to align. In contrast, however, the *SexP* gene was successfully amplified in only four out of the seven isolates analyzed, and neither intra-isolate nor inter-isolate polymorphisms were observed.

**Figure 4:**
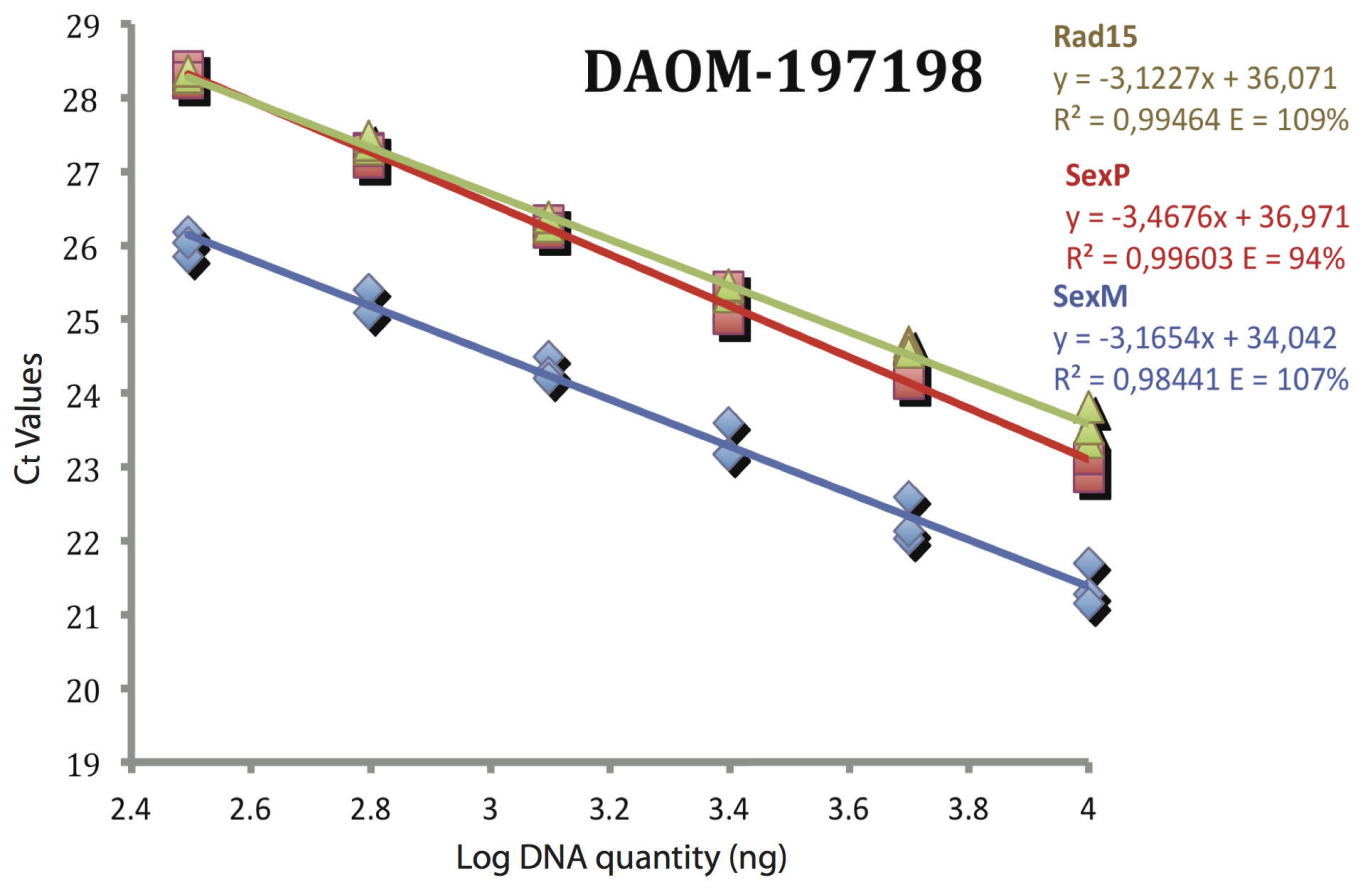
Relative quantification of SexM and SexP copy number in the isolates DAOM-197198. Results of real-time quantitative PCR show linear regressions of the cycle threshold (**C**_**t**_ values) and the log concentration of genomic DNA of *R. irregularis* isolates DAOM-197198. The analysis was performed with primers amplifying *SexM*, *SexP* and *Rad15*. For all real-time quantitative PCR experiments, three replicate amplifications for each gene were performed. Linear equations, R^2^ and PCR efficiencies are shown for each gene.

In order to investigate relative copy numbers of *SexM* versus *SexP* in the *R. irregularis* genome, we designed qPCR TaqMan assays for both mating types. The TaqMan assay for *SexM* was designed only for those alleles that showed conserved sequence portions. We compared the relative copy numbers of *SexM* and *SexP* with those obtained for the *Rad15* gene among four *R. irregularis* isolates (DAOM-197197, 234179, 234328 and 240415). The *Rad15* gene was chosen as a standard because it showed no variation in sequence both among and within isolates of *R. irregularis* [38]. In addition, *Rad15* is likely to be present in a single copy in the genome, because local Blastn search of *Rad15* sequence against *R. irregularis* genome sequence data (unpublished data) showed only one hit with the scaffold_21503. Two isolates contained both mating type transcription factors (197198 and 240415), while another two isolates (234179 and 234328) showed no evidence of *SexP*. For those isolates with *SexP*, the copy number for *SexP* was comparable to *Rad15*, although the amplification efficiencies were different between *SexP* (107%) and *Rad15* (94%). Interestingly, the number of *SexM* copies was 4-fold higher than *Rad15* with comparable amplification efficiencies (Figure 4). Based on these quantitative PCR results, we conclude that *SexM* related-genes are present in multiple copies in the *R. irregularis* genomes. 

### Sex Locus in AMF

We also tested the hypothesis that the AMF *Sex* locus may have conserved the general gene synteny found in the basal fungal lineages, i.e., a *SexM/P* flanked by TPT and RNA helicase genes. We used bioinformatic and molecular approaches to identify the genes for triose-phosphate transporters (TPT) and RNA helicases. We found 2 sequences among the contigs of 454 genomic data of 1,749 bp for TPT and 2,780 bp for RNA helicase. No contigs harbored *SexM/P* homologues and TPT or RNA Helicase coding genes. PCR followed by Sanger sequencing was used to validate those sequences. The TPT sequence contains a 106 bp intron and is transcribed based on EST data [24]. The TPT gene was found in a contig of approximately 20 kb.

**Figure 5:**
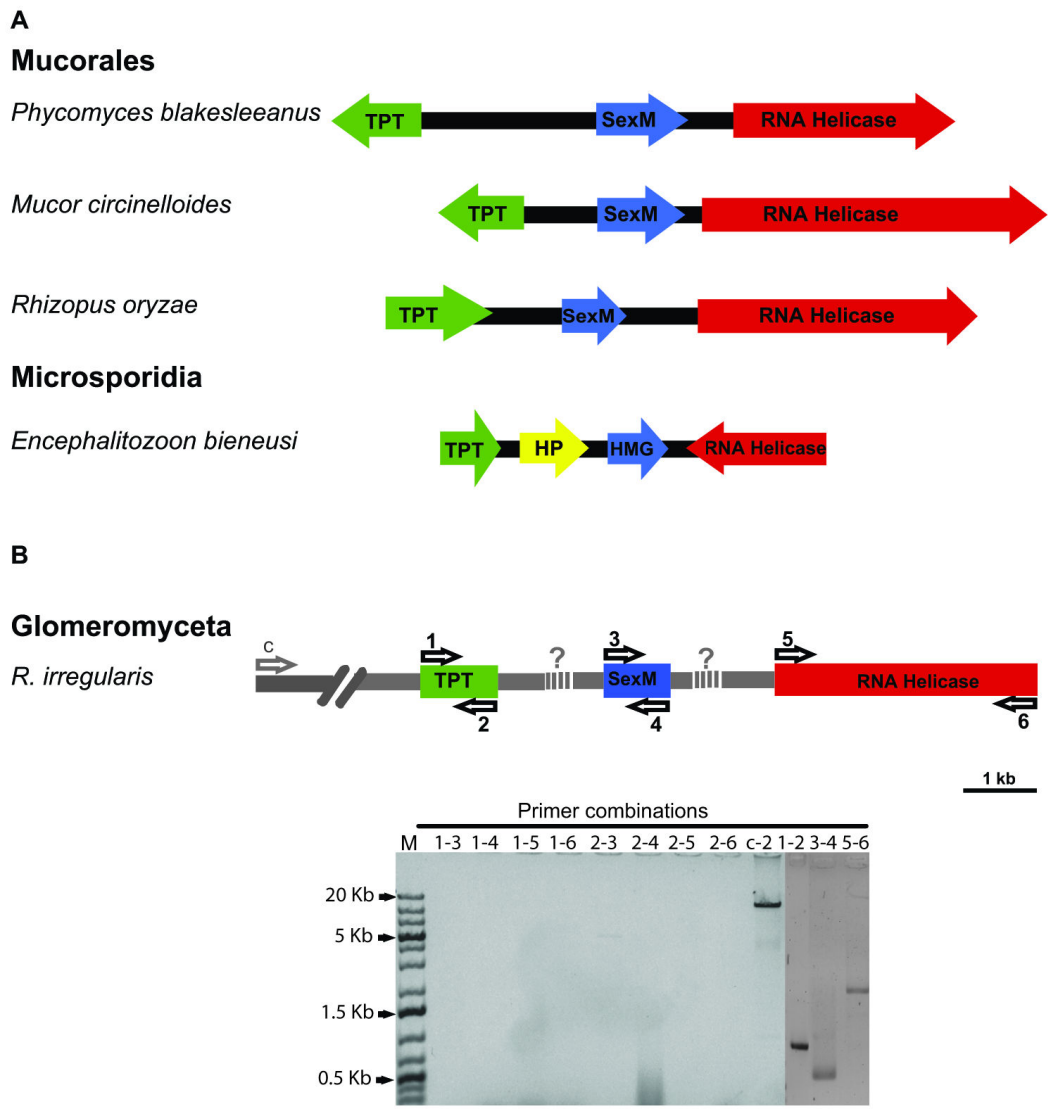
Comparison of Sex loci from Mucorales, Microsporidia and Glomeromycota. **A**, Sex locus structures of *P. blakesleeanus* (Idnurm et al., 2008), *M. circinelloides* (Lee et al., 2008), *R. oryzae* (Gryganskyi et al., 2010) and *E. bieneusi* (Lee et al., 2008) are compared with *R. irregularis* (**B**). The *TPT*, *SexM* and RNA helicase genes were found in *Rhizophagus* spp. without evidence for synteny. **B**, Six primers (1 - 6) were designed for the three genes and all combinations (eight in total) used in long PCR. No PCR amplification was observed in all combinations. Primers c (control) and 2 were used as a control for long PCR and gave a band of 19,598 pb. Combinations 1-2, 3-4 and 5-6 yielded PCR bands of the expected size, 750, 477 and 2592 bp for *TPT*, *SexM* and RNA helicase genes, respectively. M represents the size marker (1 kb plus ladder).

In order to test if the *Sex* locus in AMF is syntenic to Murorales and Microsporidia, we designed primer pairs for each of the TPT, the *SexM* transcription factor and RNA helicase genes. We tested all possible combinations considering that the orientation may differ for these genes. Figure 5A shows the arrangement for *Phycomyces blakesleeanus* [20], *Mucor circinelloides* [22], *Rhizopus oryzae* [21] and *Encephalitozoon bieneusi* (Lee et al., 2008). We tested a total of 12 different primer combinations, none of which showed a PCR band (Figure 5B, combinations 3-5, 3-6, 4-5, 4-6 not shown). However, each primer pair amplified a band of the corresponding gene with the expected size (750, 477 and 2592 bp for TPT, *SexM* and RNA helicase, respectively). Because these fragments were short, we also used two long PCR controls. The first reaction was designed to amplify a large contig of approximately 20 kb containing the gene for TPT. Primers C and 2 (Figure 5A) amplified the expected fragment of 19,598 bp. As a second control, we used mtDNA (genes and intergenic regions) that produced three PCR products all with the expected sizes, ranging between 5 and 15 kb (data not shown). Both controls showed clearly that long PCR was able to amplify large DNA fragments up to 20 kb.

## Discussion

Using genomic and transcriptomic surveys of several *Rhizophagus* spp. and *G. cerebriforme*, we clearly demonstrated the presence of conserved putative sex pheromone-sensing mitogen-activated protein (MAP) kinases comparable to those described in Ascomycota and Basidiomycota. Indeed, we find all the expected genes except for the scaffolding STE5 gene, which is also absent and not required in other fungal taxa that undergo sexual reproduction [16]. We provide evidence at the level of DNA and RNA sequences for putative genes involved in controlling sexual recognition and differentiation typical for ascomycetous yeasts and basidiomycetes. Nearly the complete sex pheromone signal transduction pathway between the STE4/STE18-dependent G-protein via the MAP kinase cascade to the nuclear STE12 transcription factor has been found in the genomes of the AMF surveyed in this study, with certain of these proteins being specific to the sexual reproduction while some genes are also involved in other cellular functions. The characterization of the proteins specifically involved in sexual reproduction, such as the pheromone receptor STE3, the pheromone transporter STE6, the alpha protein-G subunit GPA1 and the MAP Kinase FUS3 coding genes in Glomeromycota, is a strong indicator of a putative pheromone-sensing pathway in Glomeromycota, even though a sexual process has to be yet validated. Indeed, in asexually living organisms, decreased selective constraints on sex related genes should eventually result in pseudogenization and finally gene loss [40,41]. Assuming AMF to have evolved approximately 450 M years ago, the detection of well-conserved orthologs belonging to the pheromone sensing pathway strongly suggests that these genes are functional and therefore that they could be involved in sexual reproduction.

Interestingly, STE12 (*GintSTE*) has already been characterized in *R. irregularis* (formerly *G. intraradices*) and has been demonstrated to restore infectivity of a hemibiotrophic plant pathogen. It has been suggested to be a transcription factor that plays a role in early steps of root colonization by AMF [42]. Therefore, these proteins could be involved in several cellular processes, as is *S. cerevisiae* STE12. When co-activated and associated with TEC1 through the filamentation MAP Kinase pathway, STE12 activates filamentous and invasive growth [43]. Like STE12, most of the pheromone-sensing cascade components (e.g. STE7, STE11, STE20, STE50) are common with filamentation pathways. We thus highlighted the existence of several MAP Kinase cascade in AMF [17]. 

However, our approach was not able to detect pheromone peptide coding genes, although a Ste3 ortholog, coding for an a-pheromone receptor, and Ste6 gene, encoding the transporter for the hydrophobic farnesylated a-pheromone were found. This may be due to sequence divergence of those genes in comparison to the yeast genes, and/or to the short length for which local alignment algorithms are unsuitable for searching. For example, the protocol used in this study proved to be unable to find the pheromone coding genes in *Ustilago maydis* genome (data not shown). Further investigations and new methodological approaches will thus be needed to characterize the glomeromycotan pheromones and the remaining pheromone receptors.

The chemistry of pheromones is far from universal in fungi. In the Mucorales, a basal fungal lineage phylogenetically most closely related to *Rhizophagus* spp. [44-46], the apocarotenoid substances trisporic acid and many of its biosynthetic predecessors serve as mating pheromones [47,48]. In the zoosporic fungi of the genus *Allomyces*, previously included in the chytrid division and now regarded as an early-diverging group in the fungal kingdom Blastocladiomycota [49], the pheromones (sirenin and parisin) are sesquiterpenes and thus unrelated to sexual signal molecules in Dikarya and only distantly similar to mucoralean pheromones [14]. Trisporic acid is assumed to be the general pheromone for sexual communication in *Mucor* [47,48] and *Mortierella* [50] relatives. Since Glomeromycota constitutes another basal fungal lineage, phylogenetically closely related to the Mucorales [45], our first hypothesis was that AMF shared a trisporic acid pathway with Mucorales. One ortholog of the gene coding for a key enzyme of trisporic acid biosynthesis, TSP1, was found in *R. irregularis*. This enzyme, an NADP-dependent dehydrogenase, oxidizes the carotene-derived 4-dihydromethyltrisporate [39]. However, an orthology prediction using InParanoid [37] retrieved *Rhizopus oryzae* TSP1 orthologs in almost all ascomycotan genomes publically available in the database. Another TSP1 orthologs was also found in the basidiomycotan *Coprinopsis cinerea* genome. The *S. cerevisiae* TSP1 orthologous gene encodes for the GRE3 protein, an Aldose Reductase involved in sugar metabolism [51]. All fungal orthologs harbor the same protein family domain (Aldo-keto reductase PF00248) that was found in *R. oryzae* TSP1 with high sequence similarity. However, because TSP1 protein is shared between a broad range of fungal taxa, it is not necessary involved in sexual mating (except in Mucorales). Thus we cannot determine if TSP1 is involved in a communication process using trisporic acids in Glomeromycota. 

In addition to the pheromone responsive MAP Kinase cascade, sex-related HMG-box transcription factor coding genes homologous to those in the mating type locus were found in Glomeromycotan genomes. Surprisingly, although the signal transduction pathway corresponds to the STE gene system typical for asco- and basidiomycetes, the mating type locus for the *SexP* transcription factor genes resembles the mucoralean situation. Evidence for this is provided by a well supported clade containing mucoralean *SexP* and an *R. irregularis* homolog in phylogenetic analyses. Basal branches are generally poorly supported, as shown by previous phylogenetic analyses of HMG-type transcription factors [22]. However, the non *SexP*-related glomeromycotan sequences that we called *SexM*, are found clustered within two distinct groups, with the exception of one sequence from *G. cerebriforme*.

In Mucorales, both *SexM* and *SexP* genes code for completely different proteins, even though they are found at the same locus [52]. Generally, *SexM* and *SexP* loci are found in different individuals. The only exception so far is *Syzygites megalocarpus*, where both loci are in the genome at different locations, and where flanking genes for one have begun to decay into pseudogenes [53]. The observation that the gene for the assumed mating type transcription factor *SexP* is present in some isolates and absent in others of the same AMF species, renders it likely that *SexM* and *SexP* will be located in different nuclei with complementary mating type specific transcription factors. This hypothesis needs to be demonstrated experimentally, but if true, would suggest that mating behavior could be controlled by the *SexP/SexM* ratio in populations. The rarity of the *SexP* allele in the strains studied suggests that this mating type is also rare in the environment. A similar observation has been made for a different fungal species, the heterothallic yeast *Clavispora opuntiae*. In this ascomycetous yeast, mating is also a rare event, observed exclusively on a global scale [54] and never in laboratory experiments. The low frequency of *SexP* in cultivated strains could explain why sexual reproduction has not been observed *in vitro*.

Furthermore, the *SexM* copy number was observed to vary between isolates, possibly as a result of allelic sampling. However, due to the coenocytic mycelium of AMF, we cannot determine if *SexM* and *SexP* occur within one nucleus or if they reside in different nuclei in a common cytoplasm. If indeed there is only one *SexP* copy, as we found, a parsimonious solution is that *SexP* is present in each nuclei along with multiple copies of *SexM*. But if there is more than one copy, different nuclei could carry either *SexM* or *SexP*. Presently, we do not know whether AMF are genetically homothallic, pseudohomothallic or heterothallic. 

We did not find direct evidence to support synteny around the putative *Sex* locus in *R. irregularis*. Nonetheless, the lack of synteny in Glomeromycota does not necessarily rule out a sexual cycle. On the one hand, the intergenic regions between the genes for TPT, *SexM* and RNA helicase may simply exceed 20 kb in length and thus not be amplifiable by long PCR. On the other hand, it has been recently demonstrated that despite their synteny, the TPT and RNA helicase genes are not orthologous between Microsporidia and Mucorales [55], and may have diverged even before the fungal kingdom arose. This synteny should therefore be considered as a plesiomorphy, indicating that the role of these loci cannot be inferred without functional analyses.

In conclusion, we found a great diversity of *SexM* gene sequence and homogeneous *SexP* sequences. Both genes differed in terms of copy number per genome among *G. irregulare* isolates. We found no evidence for synteny of the *SexM/P* with the triosephosphate transporter gene (*TPT*) and the RNA helicase gene, although synteny at this locus may be a plesiomorphy. We also presented evidence that *R. irregularis* has almost all the functional proteins used in the pheromone signal transduction cascade in ascomycetes, including some of which completely specific to this pathway. Our results, together with previous observations on the meiotic machinery, suggest that Glomeromycota could undergo a sexual reproduction cycle. 

## Supporting Information

Figure S1 **3D structure comparison of XYL1, XYL2 and TSP1.** A - Structure alignment of cosubstrate binding amino acids of TSP1 (purple) and XYL2 (green). B - Structure alignment of active site of TSP1 (dark blue) and XYL2 (cyan).
(PDF)

Figure S2 **Phylogenetic trees of Sex pheromone-sensing pathway and Sex locus.** Maximum likelihood of amino acid sequences of pheromone-sensing pathway genes (FUS3, CDC42, STE3, STE12, STE18, STE50, GPA1, STE4, STE6, STE7, STE11, STE20, TPT and RNA Helicase genes) analyzed with the LG+G+F (with four distinct gammacategories) phylogenetic model of mating type HMG-box proteins. The *Rhizophagus irregularis* and *Glomus* *cerebriforme* sequences are highlighted in blue. The ascomycotan, basidiomycotan, as well as the human outgroup sequences are in black. The star (*) means unrooted trees.
(PDF)

Table S1 **Accession numbers of AMF genes involved in the pheromone-sensing pathway and genes orthologs with mucoralean sex-locus genes.** In sequence reference column: clarum = *Glomus* *clarum*, cereb = *Glomus* *cerebriforme*.
(DOCX)

Table S2 **Primers used to validate the sequences of genes of pheromone-sensing pathway and Sex locus using PCR and Sanger sequencing.** Primers and probes used in quantitative PCR are also listed.
(DOCX)

Table S3 **List of AMF genes involved in the pheromone-sensing pathway and genes orthologs with mucoralean sex-locus genes.**
(DOCX)

Table S4 **Similarity matrix (in %) of fungal orthologs of *Rhizopus oryzae* TSP1 protein, among 15 fungal species.**
(XLS)

Video S1 **3D structure comparison of XYL1, XYL2 and TSP1.**
(AVI)
