# Supplementary figures and images for: Mating Type Gene Homologues and Putative Sex Pheromone-Sensing Pathway in Arbuscular Mycorrhizal Fungi, a Presumably Asexual Plant Root Symbiont
Source: PLoS One. 2013 Nov 19;8(11):e80729. doi: 10.1371/journal.pone.0080729 (PMC3834313; doi:10.1371/journal.pone.0080729)

Figure S1

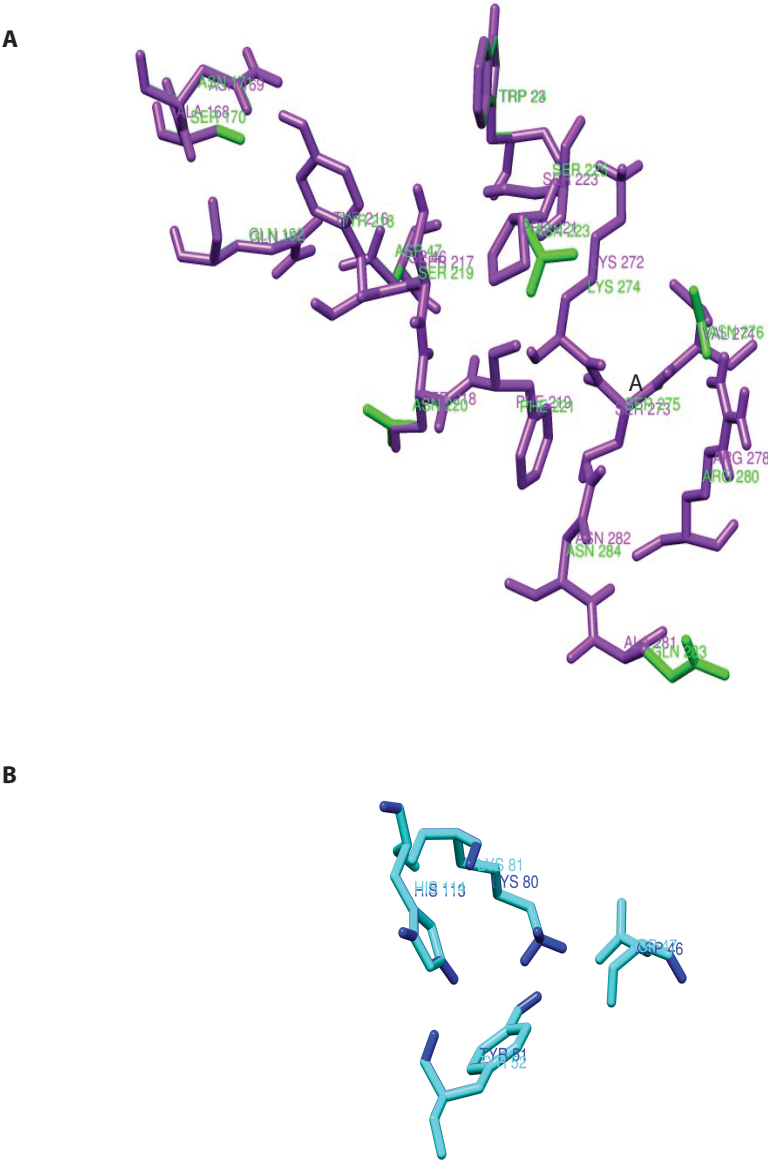

Supplement: Figure S1 — 3D structure comparison of XYL1, XYL2 and TSP1. A - Structure alignment of cosubstrate binding amino acids of TSP1 (purple) and XYL2 (green). B - Structure alignment of active site of TSP1 (dark blue) and XYL2 (cyan). (PDF) [file pone.0080729.s001.pdf]

Figure S2

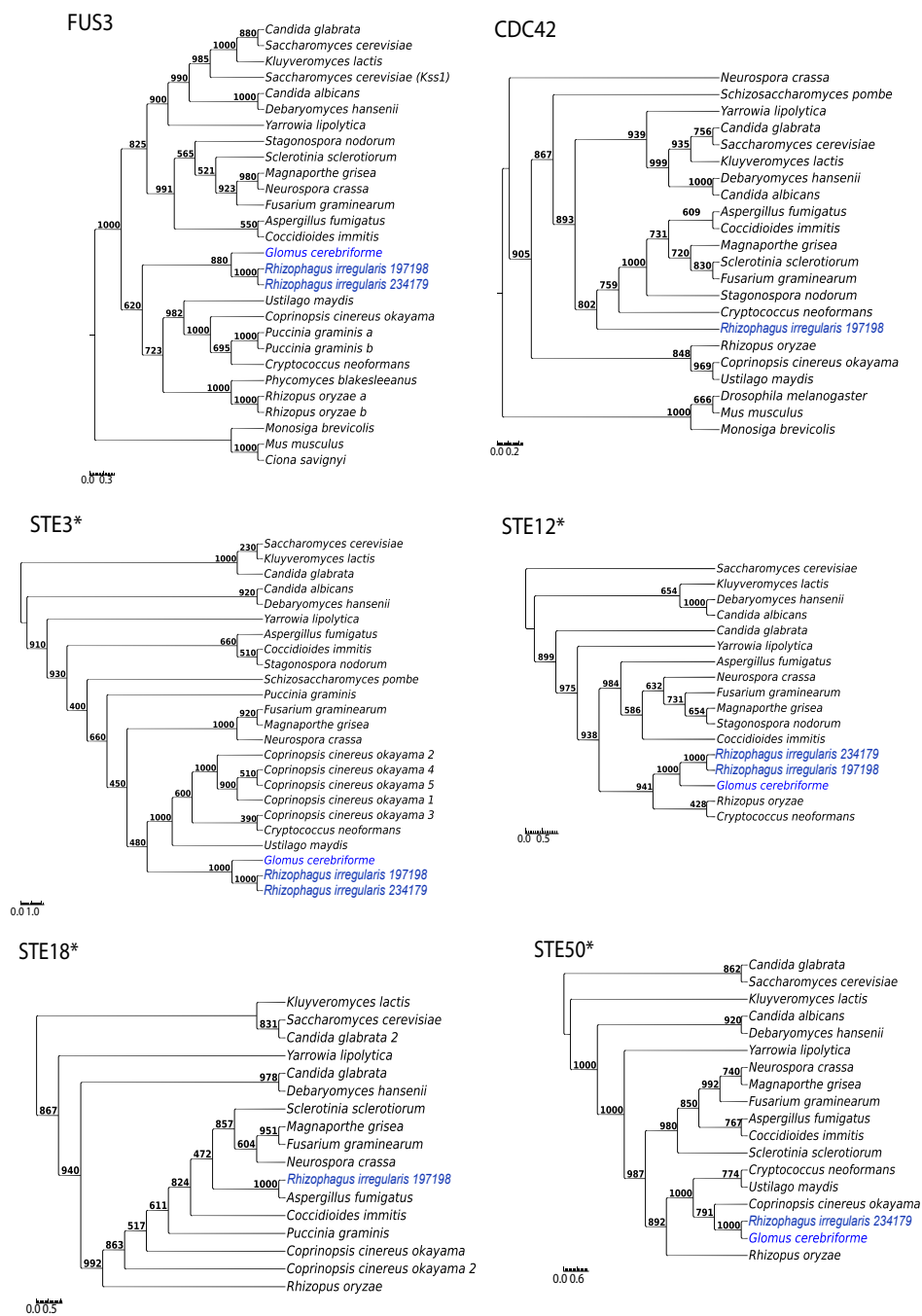

GPA1

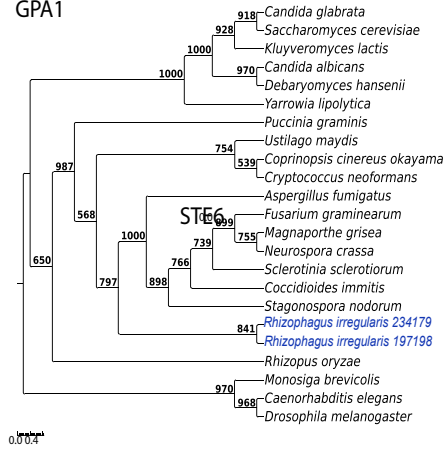

STE4

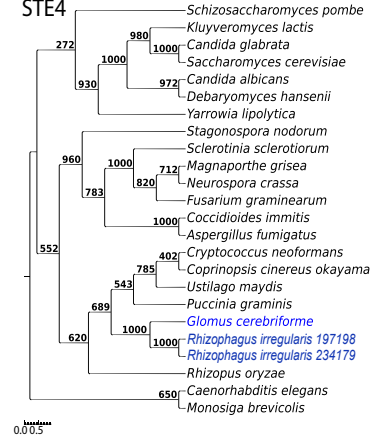

STE6

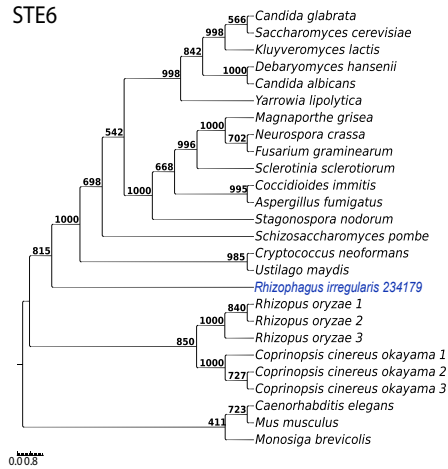

STE7

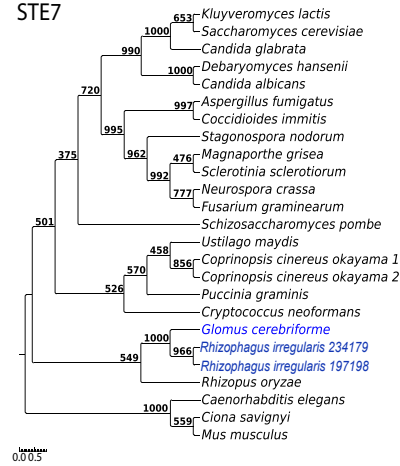

STE11

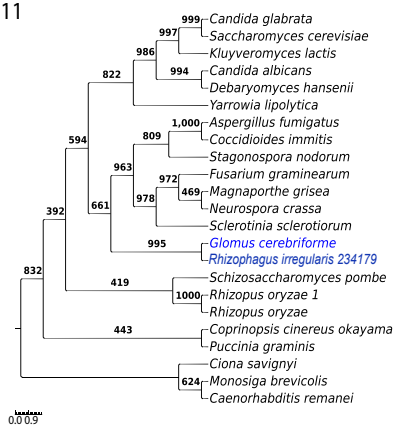

STE20

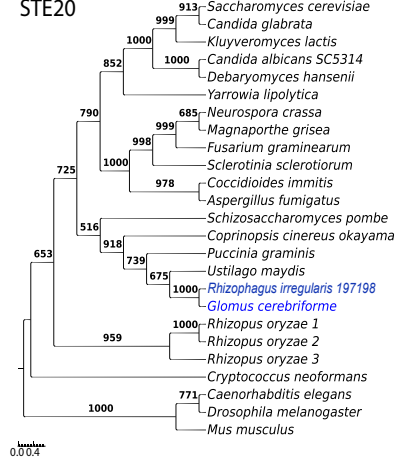

TPT

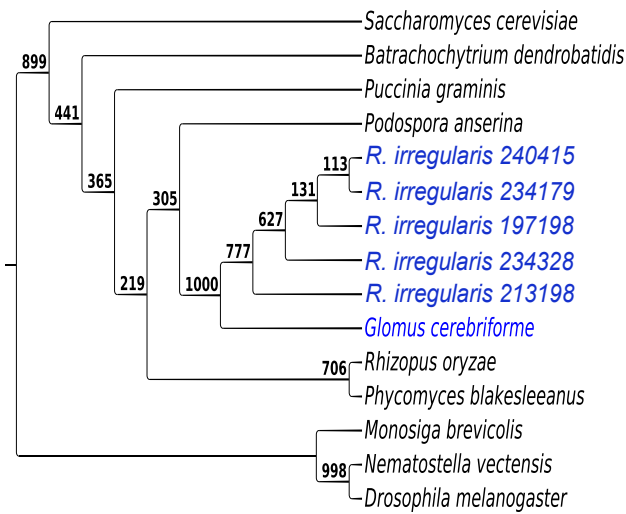

0.0 0.9

RNA helicase

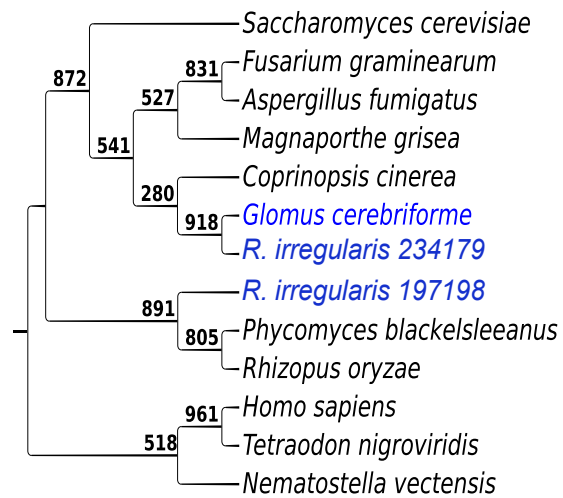

0.0 0.6

Supplement: Figure S2 — Phylogenetic trees of Sex pheromone-sensing pathway and Sex locus. Maximum likelihood of amino acid sequences of pheromone-sensing pathway genes (FUS3, CDC42, STE3, STE12, STE18, STE50, GPA1, STE4, STE6, STE7, STE11, STE20, TPT and RNA Helicase genes) analyzed with the LG+G+F (with four distinct gammacategories) phylogenetic model of mating type HMG-box proteins. The Rhizophagus irregularis and Glomus cerebriforme sequences are highlighted in blue. The ascomycotan, basidiomycotan, as well as the human outgroup sequences are in black. The star (*) means unrooted trees. (PDF) [file pone.0080729.s002.pdf]
