# Supplementary material for: Mating Type Gene Homologues and Putative Sex Pheromone-Sensing Pathway in Arbuscular Mycorrhizal Fungi, a Presumably Asexual Plant Root Symbiont
Source: PLoS One. 2013 Nov 19;8(11):e80729. doi: 10.1371/journal.pone.0080729 (PMC3834313; doi:10.1371/journal.pone.0080729)
Supplement: Table S1 — Accession numbers of AMF genes involved in the pheromone-sensing pathway and genes orthologs with mucoralean sex-locus genes. In sequence reference column: clarum = Glomus clarum, cereb = Glomus cerebriforme. (DOCX) [file pone.0080729.s003.docx]

**Table S 1** Accession numbers of AMF genes involved in the pheromone- sensing pathway and genes orthologs with mucoralean sex-locus genes. In sequence reference column : clarum = *Glomus clarum*, cereb = *Glomus cerebriforme*.

| **Protein name** | Sequence reference | Accession number |
| --- | --- | --- |
| STE6 | clarum.ste6\|contig_15988\|length_1614 | HG421031 |
|  | cereb.ste6\|contig_38722\|length_2365 | HG421032 |
| STE3 | clarum.ste3\|contig_42108 | HF679491 |
|  | cereb.ste3\|contig_41108 | HF679501 |
| GPA1 | clarum.gpa1\|contig_49568 | HF679492 |
|  | cereb.gpa1\|contig_35653 | HF679502 |
| STE4 | clarum.ste4\|contig_24821 | HF679493 |
|  | cereb.ste4\|contig_34733\|length_2644 | HF679503 |
| STE20 | cereb.ste20\|contig_11801 | HF679505 |
| STE11 | clarum.ste11\|contig_38381 | HF679495 |
|  | cereb.ste11\|contig_13823 | HF679504 |
| STE50 | clarum.ste50\|contig_1473 | HF679498 |
|  | cereb.ste50\|contig_13761 | HF679506 |
| STE7 | clarum.ste7\|contig_28835 | HF679496 |
|  | cereb.ste7\|contig_9263 | HF679507 |
| FUS3 | clarum.fus3\|contig_38756 | HF679497 |
|  | cereb.fus3\|contig_41297 | HF679508 |
| STE12 | clarum.ste12\|contig_31618 | HF679499 |
|  | cereb.ste12\|contig_25151 | HF679509 |
| TPT | cla\|contig_23207\|length_3079 | HF679510 |
|  | cer\|contig_41723\|length_1850 | HF679515 |
| Helicase | cla\|contig_32028\|length_3591 | HF679524 |
|  | cer\|contig_7335\|length_6732 | HF679525 |
| Mating type HMG-box protein | clarum_47758\|length_1193 | HF679512 |
|  | clarum_25315\|length_1226 | HF679513 |
|  | clarum_45424\|length_1634 | HF679514 |
|  | cereb_18418\|length_2966 | HF679517 |
|  | cereb_25586\|length_2745 | HF679518 |
|  | cereb_47520\|length_869 | HF679519 |
|  | cereb_7917\|length_789 | HF679520 |
|  | cereb_15789\|length_1114 | HF679521 |
|  | cereb_34789\|length_1548 | HF679522 |
|  | cereb_28710\|length_659 | HF679523 |
