# Supplementary material for: Mating Type Gene Homologues and Putative Sex Pheromone-Sensing Pathway in Arbuscular Mycorrhizal Fungi, a Presumably Asexual Plant Root Symbiont
Source: PLoS One. 2013 Nov 19;8(11):e80729. doi: 10.1371/journal.pone.0080729 (PMC3834313; doi:10.1371/journal.pone.0080729)
Supplement: Table S2 — Primers used to validate the sequences of genes of pheromone-sensing pathway and Sex locus using PCR and Sanger sequencing. Primers and probes used in quantitative PCR are also listed. (DOCX) [file pone.0080729.s004.docx]

**Table S2** Primers used to validate the sequences of genes of pheromone-sensing pathway and Sex locus using PCR and Sanger sequencing. Primers and probes used in quantitative PCR are also listed.

| **Primer name** | **5’ – 3’ sequence** |
| --- | --- |
| Nuclear_control | ACC ACT TGA TTA CCA CGC GA |
| TPT_F | ATC GAT AAG ATT GAT AAT GCT |
| TPT_R | CCG TT ACT CTT ACG TGG ATA |
| SexM_F | CCT AGA ATG TTA CCT GAT ACT G |
| SexM_R | CAC CAT CAG TAG ACA TAC GTC T |
| Helicase_F | TGA GGT TGG AAG TGC TT |
| Helicase_R | ATC TGG CAG ATG TCA AGA TCA GA |
| Ste3_F | ACA ACT CCT ANT CCT ACT CAA C |
| Ste3_R | CTA TAG CTA TTA TTG GAG GCC AC |
| Ste4_F | TGC CAA TCA TCT GCT AAT CTG AC |
| Ste4_R | CTC ATT ACA TCA CCA GTA TGG TC |
| Ste7_F | CGA ATC GTT GCG TGT GTA ACG |
| Ste7_R | CGC AAG TTA TGC ACA TTA CCA C |
| Ste11_F | tgt agc agt caa aca agt ta |
| Ste11_R | ctt cca gta tca tta gta |
| Ste20_F | CTA TTG TCA TTG CAA GGA GAT ATC |
| Ste20_R | AGC GAA TTG GAG TAT CTG GTA AC |
| Ste50_F | CCT CAA GTA ACA ATC AAT GAC ACG |
| Ste50_R | AAT CCT AAC GTA CCA ACT TGG TAC |
| Fus3_F | CAG ATA TGC ATC GTG TAA TTC GC |
| Fus3_R | GGA TTG GAT CAG CAG ATG GTT C |
| Ste12_F | CGT TCC ATG CTT CGG ATG GGC ATA |
| Ste12_R | CGT TCC ATG CTT CGG ATG GGC ATA |
| Gpa1_F | TAG GTG CTG GTG AAT CGG GC |
| Gpa1_R | AAA ACA TTC TAA TAC ACT T |
| Ste18_F | CAA CAG CAA CAG CAA CAG |
| Ste18_R | CAT AAG CGT ACA ACA AAC A |
| SexM_qpcr_probe | AAACAAAGATCAAGAAACCA |
| SexM_qpcr_F | TCCACCAAAGGCTGCATCA |
| SexM_qpcr_R | GATGAAAGCATTTGGTGGTCTTG |
| SexP_qpcr_probe | CGGATATAACTCTTCATGTTCT |
| SexP_qpcr_F | TCGCGATTTTTAAGGTTTCTCAT |
| SexP_qpcr_R | ACACGACAAAAGAAAACAAATTGC |
| Rad15_qpcr_probe | CTTGAAGTTAATCCGATTCC |
| Rad15_qpcr_F | TGACTAACTGTAGGGTGTAAGCAA AGG |
| Rad15_qpcr_R | CCCAGGGTGTGGAAGAATCA |
