# Supplementary material for: Mating Type Gene Homologues and Putative Sex Pheromone-Sensing Pathway in Arbuscular Mycorrhizal Fungi, a Presumably Asexual Plant Root Symbiont
Source: PLoS One. 2013 Nov 19;8(11):e80729. doi: 10.1371/journal.pone.0080729 (PMC3834313; doi:10.1371/journal.pone.0080729)
Supplement: Table S3 — List of AMF genes involved in the pheromone-sensing pathway and genes orthologs with mucoralean sex-locus genes. (DOCX) [file pone.0080729.s005.docx]

**Table S3** List of AMF genes involved in the pheromone- sensing pathway and genes orthologs with mucoralean sex-locus genes.
